# A Cyclized Helix‐Loop‐Helix Peptide as a Molecular Scaffold for Cell‐Membrane Permeable Inhibitors for the Interaction Between Estrogen Receptor α and Coactivator SRC1

**DOI:** 10.1002/cbic.202500232

**Published:** 2025-06-20

**Authors:** Daisuke Fujiwara, Shunsuke Inaura, Yuna Tanaka, Sayoko Ito‐Harashima, Asako Yamaguchi‐Nomoto, Masanobu Kawanishi, Takashi Yagi, Ikuhiko Nakase, Ikuo Fujii

**Affiliations:** ^1^ Graduate School of Science Osaka Metropolitan University Gakuen‐cho 1‐1 Sakai, Osaka 599‐8531 Japan; ^2^ Graduate School of Science Osaka Prefecture University Gakuen‐cho 1‐1 Sakai, Osaka 599‐8531 Japan; ^3^ Graduate School of Agriculture Osaka Metropolitan University Gakuen‐cho 1‐1 Sakai, Osaka 599‐8531 Japan

**Keywords:** cell membrane permeability, inhibitor, nuclear receptors, peptides, protein–protein interactions

## Abstract

The molecular design of inhibitors against intracellular protein–protein interactions (PPIs) is of interest for drug discovery and chemical biology. Herein, a novel cyclized helix‐loop‐helix (cHLH) peptide that inhibited the intracellular PPI between estrogen receptor alpha (ERα) and coactivator SRC1 are designed. The peptide, cHLH‐ERα, bound to ERα and inhibited the interaction between ERα and the coactivator SRC1. Cellular imaging and yeast reporter assays showed that cHLH‐ERα penetrated the cell membrane and exhibited antagonistic activity against ERα‐SRC1 to inhibit the growth of a breast cancer cell.

## Graphical abstract

A cyclized helix‐loop‐helix (cHLH) peptide is used as a molecular scaffold to generate cell‐permeable protein–protein interactions inhibitors by bifunctional grafting. The steroid receptor coactivator‐1 (SRC1) epitope (red) was grafted onto the C‐terminal helix, and six arginine residues (cyan) were grafted onto the other helix. The designed peptide cHLH‐ERα inhibited the estrogen receptor α (ERα)–SRC1 interaction and showed cell membrane permeability.

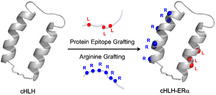

## 1 Introduction

**Figure 1:**
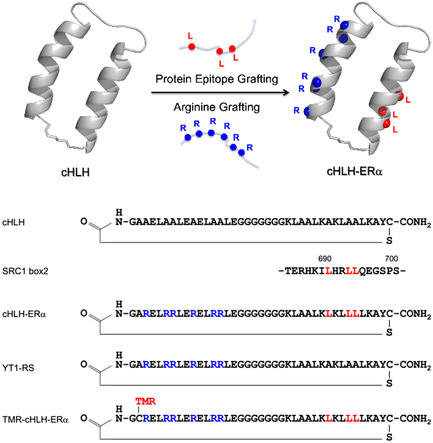
Schematic illustration of the design of the cyclized HLH (cHLH) peptide exhibiting inhibitory activity for the ERα–SRC1 interaction and cell membrane permeability. The design combines protein epitope grafting and arginine grafting. The scaffold HLH peptide YT1, the designed peptide cHLH‐ERα, the negative control peptide YT1‐RS, and the SRC1 box 2 domain with the LXXLL motif are shown. TMR: TMR.

The molecular design of inhibitors against intracellular protein–protein interactions (PPIs) is of interest in drug discovery and chemical biology.^[^ ^1^, ^2^ ^]^ We previously designed a conformationally constrained peptide with a helix‐loop‐helix (HLH) structure as a molecular scaffold for PPI‐inhibitors (**Figure** **1**).^[^ ^3^, ^4^ ^]^ Additionally, we constructed phage‐ or/and yeast‐displayed libraries and screened the libraries against targeted proteins to obtain inhibitors.^[^ ^5^, ^6^, ^7^ ^]^ Furthermore, bifunctional molecular grafting onto the cyclic HLH peptide (cHLH) provided an inhibitor against the intracellular p53‐human MDM2 (HDM2) interaction.^[^ ^8^ ^]^ In the study, arginine grafting conferred cell membrane permeability, and protein epitope grafting endowed binding activity to the target protein HDM2.^[^ ^8^ ^]^ Here, we emphasize that the bifunctional molecular grafting can be adapted easily to a variety of intracellular target proteins with the α‐helical binding epitopes to generate the PPI inhibitors.^[^ ^9^ ^]^ In this work we adopted the bifunctional grafting to generate an inhibitor for the estrogen receptor alpha (ERα)−steroid receptor coactivator‐1 (SRC1) interaction.^[^ ^10^ ^]^

ERα is a ligand‐regulated transcription factor that is a nuclear receptor (NR). NRs are important for cell proliferation, expressing the genes involved in cell growth.^[^ ^11^, ^12^, ^13^ ^]^ Several ERα‐ligand inhibitors, such as tamoxifen, have been developed to treat breast cancer. However, tamoxifen has agonistic effects on ERα in uterine cancer cells and increases the risk of endometrial cancer, highlighting the need for novel drug candidates with different mechanisms of action.^[^ ^14^, ^15^ ^]^ The binding of estrogen to ERα changes the conformation of the ligand binding domain (LBD), resulting in interaction with the translational coactivator SRC1.^[^ ^16^, ^17^, ^18^ ^]^ There are three leucine epitopes (Leu^690^, Leu^693^, and Leu^694^) on the α‐helical structure of SRC1.^[^ ^19^, ^20^, ^21^, ^22^, ^23^ ^]^ This protein–protein interaction (ERα–SRC1) provides a target interface for alternative inhibitors of the ERα–ligand interaction, which could be useful for the treatment of breast cancer. Helical peptides containing the LXXLL motif, stapled peptides and apamin mini‐proteins can inhibit ER‐transcription and hold promise as ERα‐SRC1 inhibitors.^[^ ^24^, ^25^, ^26^, ^27^, ^28^, ^29^ ^]^ However, few peptide inhibitors exhibit potent activity at the cellular level due to their low cell permeability. Herein, we report the utility of cHLH peptides as molecular scaffolds for intracellular drug design. We previously constructed a yeast reporter assay to examine antagonists for the ERα‐SRC1 interaction.^[^ ^30^ ^]^ So, here we specifically describe the generation of cHLH peptides that can cross the cell membrane and inhibit the ERα–SRC1 interaction.

## 2 Results and Discussion

The LXXLL motif of SRC1 was grafted onto the C‐terminal helix of the cHLH peptide (Figure 1) to provide binding activity towards ligand‐bound ERα. Additionally, six arginine residues were grafted onto the N‐terminal helix of the cHLH peptide to confer cell membrane permeability.^[^ ^8^ ^]^ We synthesized the designed peptide cHLH‐ERα, and the non‐binding peptide YT1‐RS to use as a negative control (Figure 1).^[^ ^8^ ^]^ The corresponding linear peptides were synthesized using a standard solid‐phase method, and then were cyclized through a thioether bond linkage between the N‐terminal *N*‐chloroacetylglycine and the C‐terminal cysteine. Circular dichroism (CD) spectrometry revealed that the high α‐helicity of the cyclic peptide cHLH‐ERα was comparable with that of the control peptide YT1‐RS, while the linear peptide HLH‐ERα possessed lower α‐helicity (Figure S4, Supporting Information). The C‐terminal fragment (14‐mer) of HLH‐ERα‐C showed a CD spectrum consistent with a random conformation (Figure S4, Supporting Information). These results suggested that the LXXLL motif grafted onto cHLH peptide was preorganized into an α‐helical conformation.

The binding activity of cHLH‐ERα was examined by fluorescence polarization (FP) analysis.^[^ ^26^, ^29^ ^]^ The cHLH peptides were labeled with tetramethylrhodamine (TMR) to provide the fluorescent cHLH peptide TMR‐cHLH‐ERα, and fluorescent TMR‐YT1‐RS as a negative control (Figure 1, Figure S2, Supporting Information). Recombinant ERα/LBD (ERα^302–552^) was prepared as previously reported, and its binding activity to SRC1 in the presence of the ligand 17*β*‐estradiol was verified by using TMR‐labeled SRC peptide (FL‐SRC1Box2), giving a *K*_D_ value of 7.8 μm (Figure S5A, Supporting Information). Peptide TMR‐cHLH‐ERα bound to ERα/LBD with a *K*_D_ value of 285 nm (Figure S5B, Supporting Information), while TMR‐YT1‐RS (Figure S2, Supporting Information) showed no binding activity. Furthermore, the FP analysis showed that peptide cHLH‐ERα inhibited the interaction between ERα/LBD (20 μm) and FL‐SRC1Box2, with an IC_50_ value of 7.1 μm (Figure S5C, Supporting Information).

**Figure 2:**
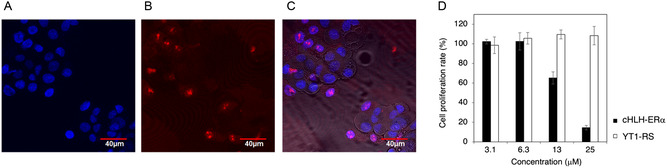
Cellular activities of the designed peptide cHLH‐ERα. Confocal microscopy images of A) MCF‐7 cells treated with Hoechst 33 342 and B) 3 μm TMR‐labeled cHLH‐ERα (TMR‐cHLH‐ERα), and C) the merged figure. D) The viability of the MCF‐7 cells treated with 3.125 to 25 μm cHLH‐ERα or with YT1‐RS peptide in the presence of 10 nm 17*β*‐estradiol, determined by using the WST‐1 assay .

The poly‐arginine induced cell membrane permeability of TMR‐cHLH‐ERα was revealed by confocal laser microscopy (**Figure** **2**A–C, Figure S6, Supporting Information). MCF‐7 breast cancer cells were treated with 3 μm TMR‐cHLH‐ERα peptide at 37 °C. As shown in Figure 2, TMR‐cHLH‐ERα appeared diffuse inside the cells and partially accumulated inside the nuclei stained with Hoechst 33 342 (Figure 2C), and the TMR‐labeled negative control peptide TRM‐YT1‐RS showed cell membrane permeability by confocal laser microscopy both at 37 °C and 4 °C (Figure S6, S7, Supporting Information), speculating that the cHLH peptides were internalized into MCF‐7 cells probably by a temperature‐independent mechanism as reported elsewhere.^[^ ^31^ ^]^ These results suggested that cHLH‐ERα can inhibit ERα–SRC1 interaction inside cells, leading to inhibition of cancer cell proliferation.

Next, we examined the inhibitory activities of the peptides for the 17*β*‐estradiol‐dependent proliferation of MCF‐7 breast cancer cells using the WST‐1 assay (Figure 2D). The MCF‐7 cells were incubated with the serially diluted peptides cHLH‐ERα or YT1‐RS in the presence of 10 nM 17*β*‐estradiol. The cHLH‐ERα peptide inhibited the proliferation of MCF‐7 cells dose‐dependently, while the negative control YT1‐RS showed no inhibitory activity. These results suggested that the cHLH‐ERα peptide inhibited ERα–SRC1 interactions inside the cells, thereby inhibiting cancer cell proliferation.

**Figure 3:**
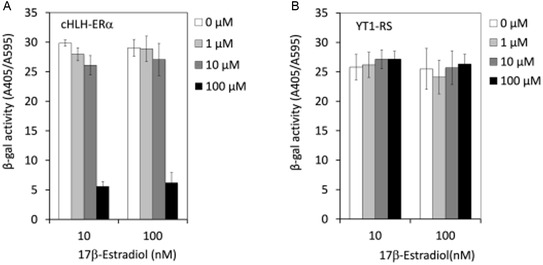
The yeast reporter assay, used to examine the A) 17*β*‐estradiol‐dependent antagonistic activity of cHLH‐ERα and B) that of the negative control YT1‐RS. This assay examines the antagonistic activity of the tested molecules for ERα, based on the decrease in *β*‐galactosidase activity by antagonists.

We then confirmed that cHLH‐ERα directly inhibits the interaction between ERα and SRC1 in cells by using a yeast reporter assay previously constructed using the *Saccharomyces cerevisiae* strain cwp1Δcwp2Δ as a host.^[^ ^30^ ^]^ The expression plasmids for human ERα and the transcriptional coactivator SRC1 were transformed, along with the *lacZ* reporter plasmid carrying the estrogen response element (RE).^[^ ^13^ ^]^ The production of ERα and SRC1 was regulated by the galactose‐inducible *GAL1‐10* promoter. In response to 17*β*‐estradiol, ERα, and SRC1 formed a complex on the RE, inducing the ligand‐dependent expression of *β*‐galactosidase (Figure S8, Supporting Information). The genes encoding the major cell wall mannoproteins Cwp1p and Cwp2p were deleted,^[^ ^30^ ^]^ probably allowing the cHLH peptides to access the cell membrane by passing through the less dense cell wall of the yeast strain.^[^ ^32^ ^]^ The effect of the cHLH peptide on the ERα–SRC1 interaction was examined in the presence of 17*β*‐estradiol (10 and 100 nm). Treating the yeast cells for 18 h with the cHLH peptides indicated that 100 μm cHLH‐ERα significantly inhibited transcriptional activity (**Figure** **3**A), while the negative control peptide YT1‐RS showed no inhibitory activity (Figure 3B). The cHLH peptides showed no cytotoxicity against the yeast strain at 100 μm (Figure S9, Supporting Information). These results suggested that cHLH‐ERα can penetrate the eukaryotic cell membrane and shows antagonistic activity against the ERα–SRC1 interaction inside yeast cells.

## 3 Conclusion

Here, we newly designed a cell membrane‐permeable cHLH peptide inhibitor for the ERα–SRC1 interaction. The peptide bound to the ERα and inhibited the ERα–SRC1 interaction. The antagonistic activity against the ERα was detected by the yeast reporter assay, and we speculated that the peptide passed through not only the cell wall but also the cell membrane. Confocal laser microscopy with TMR‐labeled cHLH‐ERα revealed the cell membrane permeability of cHLH‐ERα for MCF‐7 cells, suggesting the membrane permeability of non‐labeled cHLH‐ERα. The peptide cHLH‐ERα inhibited the ERα–SRC1 interaction in which LXXLL motif is well conserved among coactivators, hence, the cHLH peptide could be a scaffold for designing inhibitors not only for ERα but also the other NRs.^[^ ^33^ ^]^ Further affinity maturation of cHLH‐ERα for a specific NR could optimize the peptide to develop specific inhibitor and therapeutic leads^[^ ^11^, ^12^, ^13^ ^]^ and the design of novel inhibitors against other intracellular PPIs using α‐helical binding motifs.^[^ ^9^ ^]^

## 4 Experimental Section

4.1

4.1.1

##### Reagents

Fmoc‐L‐amino acids were purchased from Novabiochem. *N*‐chloroacetyl glycine, piperidine were purchased from SIGMA. Acetic anhydride, diethyl ether, trifluoroacetic acid (TFA), and Silver trifluoromethanesulfonate (AgOTf) were purchased from nacalai tesque. *O*‐benzotriazole‐*N*,*N*,*N*,*N*'‐tetramethyl‐uronium‐hexafluorophosphate (HBTU), *O*‐(6‐Chloro‐1H‐ benzotriazol‐1‐yl)‐*N*,*N*,*N*',*N*'‐tetramethyluronium hexafluorophosphate (HCTU), *N*,*N*‐diisopropyl ethylamine (DIEA), and Fmoc‐PAL‐PEG‐PSresin (0.21 mmol g^−1^) were purchased from Watanabe Chemical Ind. The α‐cyano‐4‐hydroxycinnamic acid (CCA) was purchased from Bruker Daltonics. 5‐TMRIA single isomer was purchased from Life Technologies.

##### Peptide Synthesis

All the peptides were synthesized as described.^[^ ^8^ ^]^ Peptides were synthesized by standard solid‐phase peptide synthesis (SPPS) with Fmoc amino acids by using an automated solid phase peptide synthesizer (PSSM‐8, Shimadzu) or by using a set of equipment containing a mixer (EYELA), a vacuum pump (KNF Lab), and a tube (HiPep Laboratories). 0.1 mmol of Fmoc‐PAL‐PEG‐PS resin was deprotected with 20% piperidine/DMF v/v and washed with DMF. 0.5 mmol of Fmoc‐Amino acid was activated with HBTU or HCTU and added to the deprotected Fmoc amide resin. The subsequent elongation of peptides was performed in the same way, and the synthesized peptide was cleaved with trifluoroacetic acid (TFA) containing scavengers. The cleaved peptide was filtered, and the flow‐through was centrifuged in chilled diethylether, and then dried. The peptides were purified with reversed‐phase high‐performance liquid chromatography (RP‐HPLC) (C18, 250 × 10 mmI.D., 5 μm, YMC) with a linear gradient of water containing 0.1% TFA and acetonitrile at a flow rate 3 mL min^−1^ by using an HPLC system (Hitachi). The synthesized peptides were identified by matrix‐assisted laser desorption ionization time‐of‐flight mass spectrometry (MALDI‐TOF‐MS) (Autoflex II, Bruker Daltonics) using CCA in acetonitrile/0.1% v/v TFA (1:2) as a matrix. The peptide purities were determined with analytical HPLC (C18, 250 mm × 4.6 mmI.D., 5 μm, YMC), and verified over 90% in all peptides (Figure S3, Supporting Information).

To synthesize cyclized HLH (cHLH) peptides cHLH‐ERα, *N*‐chloroacetyl peptides containing a cysteine at the C‐terminus were synthesized by standard SPPS (*N*‐ClAcGly‐ARELRRLERELRRLEGGGGGGGKLAALKLKLLLLKAYC‐CONH_2_) and purified by RP‐HPLC and then lyophilized. The purified linear peptides were dissolved in 0.1 m NaHCO_3_ and then stirred for 5–12 h at room temperature to cyclize form forming intramolecular thioether bond. After lyophilization, the peptides were purified by RP‐HPLC as described.^[^ ^8^ ^]^ In addition, the derivatives of the peptide cHLH‐ERα were synthesized (Figure S1, Supporting Information). MALDI TOF‐MS (m/z): cHLH‐ERα calcd. 4277.1 [M + H]^+^, found 4276.1 [M + H]^+^; HLH‐ERα calcd. 4075.9 [M + H]^+^, found 4075.9 [M + H]^+^; HLH‐ERα‐1C calcd. 1698.2 [M + H]^+^, found 1698.2 [M + H]^+^.

To prepare TMR‐labeled cHLH peptides TMR‐HLH‐ERα (Figure 2) and TMR‐YT1‐RS (Figure S2, Supporting Information) in which the N‐terminal Ala was substituted with the S‐acetamidomethy (Acm) cysteine for TMR‐cHLH‐ERα (*N*‐ClAcGly‐C(Acm)RELRRLERELRRLEGGGGGGGKLAALKLKLLLLKAYC ‐CONH_2_) and TMR‐YT1‐RS (*N*‐ClAcGly‐C(Acm)RELRRLERELRRLEGGGGGGGKLAALKAK LAALKAYC‐CONH_2_), which was subsequently cyclized as described above. AgOTf was added to the peptide dissolved in TFA/Thioanisole and stirred for 1.5 h at 4 °C.^[^ ^34^ ^]^ After filtration and purification using RP‐HPLC, the purified peptide was dissolved in phosphate‐buffered saline (PBS) with 5‐TMRIA and stirred 3 h, and then the product was purified by RP‐HPLC, and lyophilized. MALDI TOF‐MS (m/z): TMR‐cHLH‐ERα: calcd. 4749.7. [M + H]^+^, found 4749.6 [M + H]^+^; TMR‐YT1‐RS: calcd. 4623.4 [M + H]^+^, found 4620.5 [M + H]^+^.

##### Live Cell Confocal Microscopy

MCF‐7 was purchased from ATCC. DMEM, and RPMI 1640, fetal bovine serum (Gibco) were purchased from Gibco. MCF‐7 cells were cultured (20 000 cells/well; 100 μL/well) in the 4‐well type 35‐mm glass‐bottom dish (Matsunami) for 24 h. 100 μL of 6 μm TMR‐labeled peptides TMR‐cHLH‐ERα and TMR‐YT1‐RS dissolved in the culture media were respectively added to the cells (final concentration: 3 μm). After 5‐min incubation at 37 °C under 5% CO_2_ atmosphere, the fluorescent images of the cells were acquired with Olympus FV1200 (Figure 2C–E, S7, Supporting Information) after the replacement of the medium to freshly prepared 100 μL medium which was pre‐warmed at 37 °C. In the cases of treatment of the cells at 4 °C (Figure S8, Supporting Information), after the 24‐h incubation, the dishes were prechilled on ice and the medium was replaced to fresh medium pre‐chilled at 4 °C.

##### Cell Viability Assay

For cell viability assay,^[^ ^35^ ^]^ ER‐positive MCF‐7 cancer cells were grown in the recommended media Dulbecco's modified Eagle medium (DMEM) supplemented with 10% heat‐inactivated fetal bovine serum and with 1% penicillin–streptomycin (Gibco) in a humidified environment with 5% CO_2_. These cells were incubated in 96‐well plates (Corning) at 5,000 cells/well and cultured for 24 h. The cells were treated with 10 nm 17*β*‐estradiol for 1 h, then a various concentrations of peptides were added. After culturing the cells for 72 h, the proliferation f cells was measured by using WST‐1 reagent (Roche) according to the standard protocols. The absorbance was measured by using a microplate reader (Model 680, Bio‐Rad). Reliability of the cell viability assay was confirmed by using Tamoxifen (nacalai tesque) as a control.

##### Yeast Reporter Assay

As previously reported,^[^ ^30^ ^]^ the yeast strain ERα cwp1Δcwp2Δ was cultured on agar plates with synthetic dextrose (glucose) complete (SDC) dropout medium lacking tryptophan, leucine, and uracil (SDC‐Trp, Leu, Ura; 2% glucose, 2% agar) for single colony isolation. The yeast colonies were grown overnight at 30 °C in SDC‐Trp/Leu/Ura/Tyr/Phe liquid medium, and the optical density at 595 nm of each culture was adjusted to 1.0 with the same medium. One μL of peptide (cHLH‐ERα or YT1‐RS) dissolved in sterile Milli‐Q water, 1 μL of 17*β*‐estradiol (dissolved in DMSO), 10 μL of the yeast culture, and 90 μL of synthetic galactose complete dropout medium SGC‐Trp/Leu/Ura/Tyr/Phe (1% galactose) was mixed in 96‐well polystyrene microplate, and the plate was sealed with tape. After incubating the plate for 18 h at 30 °C, 5 μL of yeast cell suspension was transferred to another 96‐well microplate and 100 μl of Z‐buffer (60 mm Na_2_HPO_4_, 40 mm NaH_2_PO_4_, 1 mm MgSO_4_, 10 mm KCl, 2 mm DTT, and 0.2% *N*‐lauroylsarcosine, adjusted to pH 7.0), containing 1 mg mL^−1^ *o‐*nitrophenyl‐*β*‐D‐galactopyranoside (ONPG), was added to the plates with subsequent incubation at 37 °C (until yellow color develops). Absorbance at wavelengths of 405 and 595 nm was measured using Model 680 Microplate Reader (BioRad Lab., Inc.) to estimate the *β*‐galactosidase activity as the amount of *o*‐nitrophenol (ONP) produced and yeast cell density, respectively. The relative *β*‐galactosidase activity in each well was calculated as A405/A595. In order to examine whether cHLH peptide exert cytotoxic effect on yeast growth, relative cell growth rate (% CT) was calculated using the formula: [OD_595_ (18 h)–OD_595_ (0 h) in peptide exposed wells]/[OD_595_ (18 h) ‐ OD_595_ (0 h) in solvent control wells] × 100. 17*β*‐Estradiol was added at a final concentration of 10 nm.

## Conflict of Interest

The authors declare no conflict of interest.

## Supporting information

Supplementary Material

## Data Availability

The data that support the findings of this study are available from the corresponding author upon reasonable request.
