# Supplementary material for: A Cyclized Helix‐Loop‐Helix Peptide as a Molecular Scaffold for Cell‐Membrane Permeable Inhibitors for the Interaction Between Estrogen Receptor α and Coactivator SRC1
Source: Chembiochem. 2025 Jun 20;26(13):e202500232. doi: 10.1002/cbic.202500232 (PMC12247020; doi:10.1002/cbic.202500232)
Supplement: Supplementary file 1 — Supplementary Material [file CBIC-26-e202500232-s001.pdf]

Table of Contents

1. Protein Expression and Purification

2. Peptide Synthesis

3. Circular Dichroism (CD) Spectra

4. Fluorescence Polarization

5. Live Cell Confocal Microscopy

6. Yeast Reporter Assay

7. References

1. Protein Expression and Purification

The plasmid encoding the LBD domain of ERα (ERα$^{302-552}$), was prepared from the plasmid pF1KB7810 (Kazusa DNA Res. Inst.) encoding the full length of Estrogen Receptor α as described by using pET-28a(+)vector [1]. The DNA fragment encoding ERα$^{302-552}$ was amplified by using the following two primers (ERαLBD Forward primer and ERαLBD Reverse primer), digested by the restriction enzymes BamHI and SacI, and ligated into pET-28a(+) which was digested by the same restriction enzymes. After cloning and purification of the plasmid of the ERα-LBD, the *Escherichia coli* competent cells BL21-CodonPlus-RP Competent Cell Correct Codon Bias (Agilent) were transformed by the plasmid pET-28a(+)-ERα-LBD. The transformed *E. coli* cells were grown in 2×YT containing Kanamycin (20 μg/mL) at 37 °C to an OD_{600} of 0.65. After chilling the culture at 16 °C, it was shaked with 0.5 mM IPTG at 16 °C and overnight. The cells were harvested by centrifugation and the pellets were resuspended in 25 mL of binding buffer (20 mM Tris-HCl (pH 8.0), 300 mM NaCl, 20 mM imidazole), and were sonicated and centrifuged for 30 in at 9,000 g at 4 °C. Two mL of pre-equilibrated Ni Sepharose High Performance (GE healthcare) was added to the supernatant and incubated for 1 hr on ice. The resin was washed with 20 column volume (CV) of binding buffer, and elution was carried out with 5 mL of 500 mM imidazole Buffer [20 mM Tris-HCl (pH 8.0), 500 mM NaCl, 300 mM Imidazole]. The eluate was dialyzed in 1 L of 25 mM phosphate buffer (pH 6.7), and was condensed by a centrifugal filter Amicon Ultra-4 10K.

Oligonucleotides:

ERαLBD Forward primer 5′-CGCGGATCCAAGAAGAACAGCCTGGCCTTG-3′

ERαLBD Reverse primer 5′-CCGGATGAGCTCTTAAGTGGGCGCATGTAGGCGG-3′

2. Peptide Synthesis

HLH-ERα	H_2N-ARELRRLERELRRLEGGGGGGGKLAALKLKLLLLKAY-$CONH_2$
HLH-ERα-C	H_2N-KLAALKLKLLLLKAY-$CONH_2$

Fig. S1 The derivatives of peptide cHLH-ERα: HLH-ERα and HLH-ERα-C.

TMR-YT1-RS

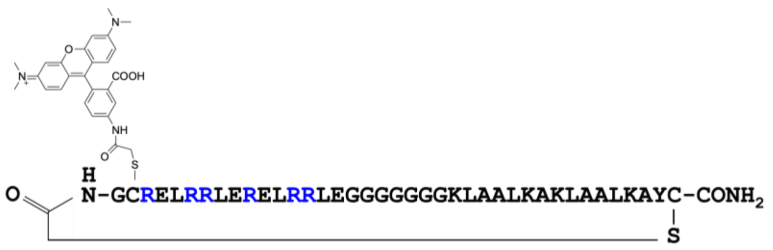

Fig. S2 Tetramethylrhodamine-labeled cHLH peptide TMR-YT1-RS.

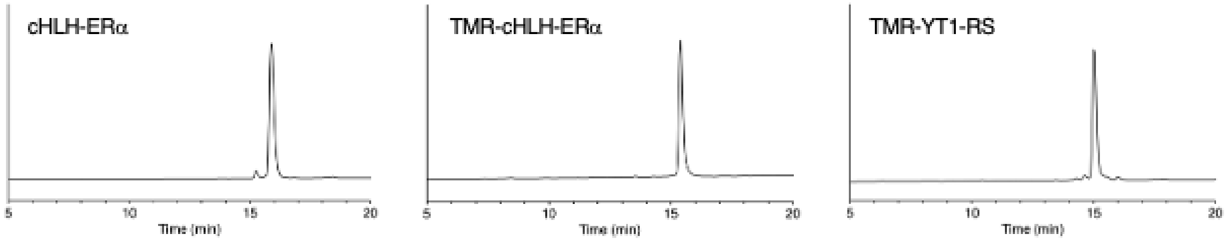

Fig. S3 HPLC profiling of the representative cHLH peptides reported in this study: cHLH-ERα, TMR-cHLH-ERα, and TMR-YT1-RS. Analytical HPLC with UV detection at 220 nm was performed with 22–42% elution with acetonitrile including 0.08% TFA for cHLH-ERα, 25–45% for TMR-cHLH-ERα, and 23–43% for TMR-YT1-RS over 20 minutes.

3. Circular Dichroism (CD) Spectra

CD spectra were recorded in 20 mM Tris-HCl (pH 8.0), 25 mM NaCl at overall peptide concentration of 20 μM on a Jasco J-820 spectrometer at 20 °C (PTC-423L thermostat, Jasco). The peptide concentrations were determined by measuring absorbance of aromatic residues [2]. Spectra were collected from 260 to 190 nm every 0.2 nm.

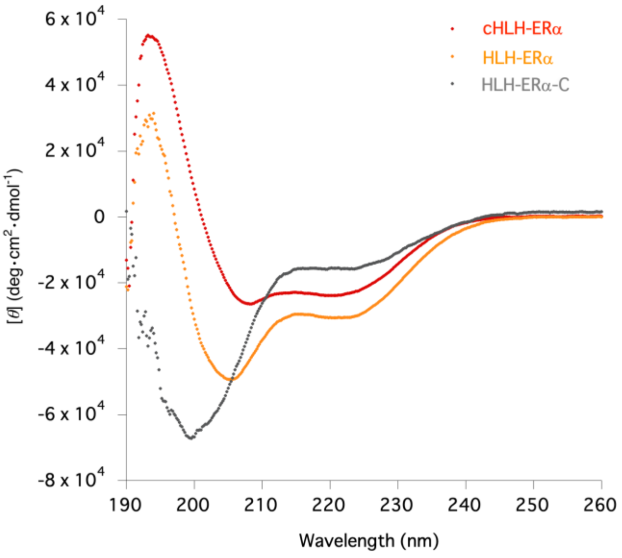

Fig. S4 The CD spectra of cHLH-ERα and the derivatives, HLH-ERα and HLH-ERα-C.

4. Fluorescence Polarization

The binding activity of the ERα-LBD was examined with 20 nM FL-SRC1Box2 (CH_3CO-C(FL)QLLTERHKILHRLLQEGSPSD-NH_2; FL: Fluorescein) with 10 μM 17β-estradiol in 20 mM Tris-HCl (pH 8.0), 25 mM NaCl, 10%(v/v) Glycerol, 1 mM TCEP as described [3]. The concentration of FL-SRC1Box2 labeled with Fluorescein and TMR-cHLH-ERα and YT1-RS were determined from the absorption of Fluorescein or Tetramethylrhodamine. The respective peptide was incubated with ERα-LBD for 30 min in 20 mM Tris-HCl (pH 8.0), 25 mM NaCl with 1 mM 17β-estradiol, and the mixture was transferred into a 384-well microtiter well (Non-binding, Black, greiner bio-one) and the fluorescence polarization was measured on a spectrofluorometer (SpectraMax iD5, Molecular Devices) at 20 °C. The K_D values were analyzed via a quadratic binding equation (Fig S5A, S5B) with the data fit to calculate by using KaleidaGraph 4.0 as described elsewhere [4]. The IC_{50} value was estimated as 7.1 μM derived from four parameter logistic equation (Fig. S5C).

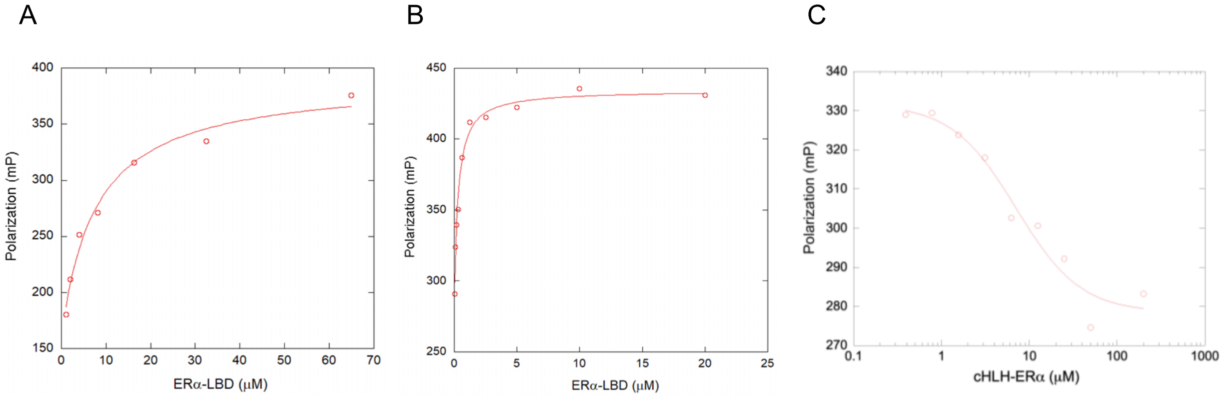

Fig. S5 The binding activity of the fluorescently-labeled peptides (A) FL-SRC1Box2 (B) TMR-cHLH-ERα to ERα-LBD, and (C) the inhibitory activity of cHLH-ERα against the ERα-LBD–FL-SRC1Box2 interaction.

5. Live Cell Confocal Microscopy

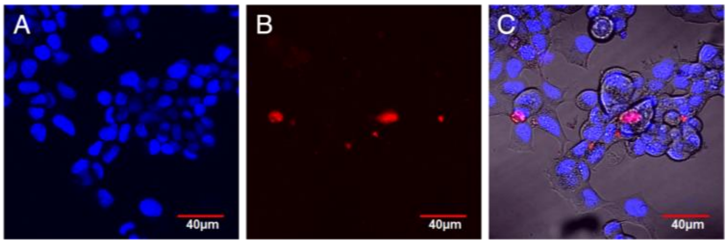

Fig. S6 Confocal microscopy images of MCF-7 cells treated with Hoechst 33342 (A) and 3 μM TMR-YT1-RS (B), and the merged figure (C) at 37 ºC.

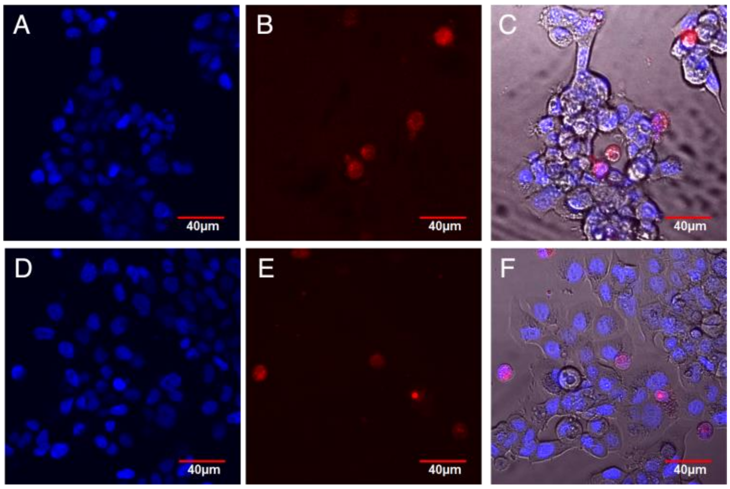

Fig. S7 Confocal microscopy images of MCF-7 cells treated with Hoechst 33342 (A, D) and 3 μM TMR-cHLH-ERα (B), 3 μM TMR-YT1-RS (E), and the respective merged figures (C, F) at 4 ºC.

6. Yeast Reporter Assay

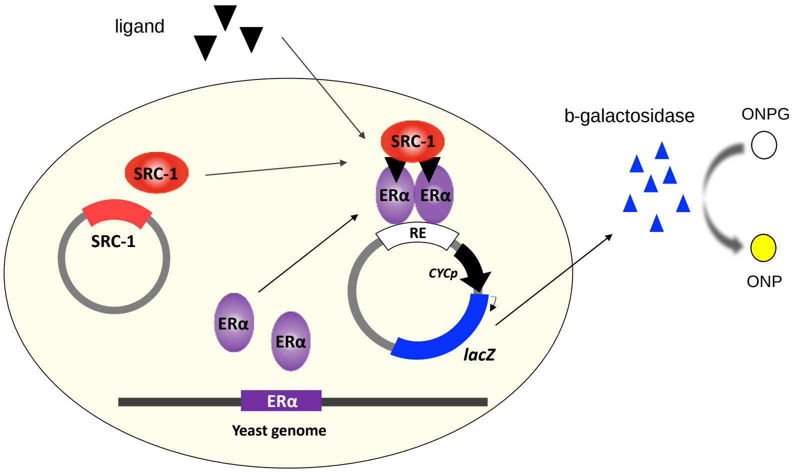

Fig. S8 Schematic illustration of the yeast reporter assay.

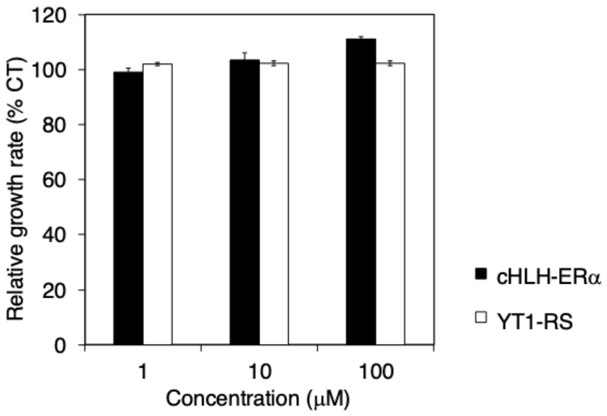

Fig. S9 The cytotoxic effect of cHLH peptides for the yeast strain used in this study.

7. References

1. S. Eiler, M. Gangloff, S. Duclaud, D. Moras, M. Ruff, *Protein Expr. Purif.* **2001**, *22*, 165–173.

2. C.N. Pace, F. Vajdos, L. Fee, G. Grimsley, T. Gray, *Protein Sci.* **1995**, *4*, 2411–2423.

3. T. Phan, H.D. Nguyen, H. Göksel, S. Möcklinghoff, L. Brunsveld, *Chem. Commun.* **2010**, *46*, 8207–8209.

4. S.C. Zondlo, A.E. Lee, N.J. Zondlo, *Biochemistry*, **2006**, *45*, 11945–11957.
